# PMLPR: A novel method for predicting subcellular localization based on recommender systems

**DOI:** 10.1038/s41598-018-30394-w

**Published:** 2018-08-13

**Authors:** Elnaz Mirzaei Mehrabad, Reza Hassanzadeh, Changiz Eslahchi

**Affiliations:** 1 Department of Computer Science, Faculty of Mathematical Sciences, Shahid Beheshti University, Tehran, Iran; 2 Department of Engineering Sciences, Faculty of Advanced Technologies, University of Mohaghegh Ardabili, Namin, Iran; 3 Department of Bioinformatics, Faculty of Computer Engineering and Information Technology, Sabalan University of Advanced Technologies (SUAT), Namin, Iran; 4 School of Biological Science, Institute for Research in Fundamental Sciences (IPM), Tehran, Iran

## Abstract

The importance of protein subcellular localization problem is due to the importance of protein’s functions in different cell parts. Moreover, prediction of subcellular locations helps to identify the potential molecular targets for drugs and has an important role in genome annotation. Most of the existing prediction methods assign only one location for each protein. But, since some proteins move between different subcellular locations, they can have multiple locations. In recent years, some multiple location predictors have been introduced. However, their performances are not accurate enough and there is much room for improvement. In this paper, we introduced a method, PMLPR, to predict locations for a protein. PMLPR predicts a list of locations for each protein based on recommender systems and it can properly overcome the multiple location prediction problem. For evaluating the performance of PMLPR, we considered six datasets RAT, FLY, HUMAN, Du *et al*., DBMLoc and Höglund. The performance of this algorithm is compared with six state-of-the-art algorithms, YLoc, WOLF-PSORT, prediction channel, MDLoc, Du *et al*. and MultiLoc2-HighRes. The results indicate that our proposed method is significantly superior on RAT and Fly proteins, and decent on HUMAN proteins. Moreover, on the datasets introduced by Du *et al*., DBMLoc and Höglund, PMLPR has comparable results. For the case study, we applied the algorithms on 8 proteins which are important in cancer research. The results of comparison with other methods indicate the efficiency of PMLPR.

## Introduction

Sub-Cellular Location (SCL) prediction of a protein is a substantial problem in Bioinformatics, because there is a close relationship between the SCL of a protein and its function^1^. Moreover, accurate prediction of subcellular localization helps to identify the potential molecular targets for drugs^2^. Furthermore, protein SCL has an important role in many other fields such as genome annotation, cytobiology and proteomics^1^. Today, protein data banks are growing rapidly, demanding fast and accurate tools for identifying the SCLs of new proteins.

Generally, there are two approaches for the protein subcellular localization problem: experimental methods and computational methods. Several experimental approaches such as green fluorescent protein^3^, microscopic detection^4^ and subcellular proteomics^5^ have been already introduced to identify subcellular locations of a protein. Unfortunately, experimental methods are time consuming and costly. That is why a large information gap exists between protein sequences and their location, and the gap grows by the day. Consequently, various computational methods have been developed to fill this gap^6–11^.

Computational methods have their advantages and disadvantages. These methods outperform experimental methods, both in terms of time and cost, but they may not be as accurate as experimental methods. Moreover, most of these computational methods focus on the single site SCL of a protein whereas the experimental researches show that many proteins are located in several subcellular locations^11^. On the other hand, most of these methods are developed for particular proteins or species^6,7,12–14^. Hence, it seems that a more comprehensive method is desired to predict multiple locations for various proteins while remaining applicable to different species.

Subcellular location prediction methods need a reliable protein-location dataset to learn their system and to evaluate their algorithm. Some computational algorithms provide improved SCL prediction by using GO information^1,15,16^. GO is a bioinformatics tools to unify gene and gene products across all species. In fact, GO provides an ontology of predefined terms covering three domains that includes cellular component, molecular function and biological process^17^. UniProtKB/Swiss-Prot is also a database which is used in many computational algorithms. The Universal Protein Resource, UniProt, is a comprehensive, knowledgebase database of protein information which includes protein sequences and functional annotations. One of the main parts of UniProt is UniProtKB repository. UniProtKB is subtended by two sections: UniProtKB/Swiss-Prot which contains the manually annotated protein location and reviewed entries, and UniProtKB/TrEMBL which consists of automatically annotated protein location and non-reviewed information^18,19^.

On the other hand, proteins within a cell do not work independently and interact with different proteins. The physical interactions between a pair of proteins imply that the physical distance between interacting proteins is very close, and so the interacting proteins tend to localize within the same subcellular compartments^20,21^. The fact that interacting proteins may share at least one location has been validated by Jiang *et al*.^22^. Therefore, protein-protein interaction information could be useful in predicting protein subcellular locations and several methods have been developed based on protein-protein interactions to predict protein subcellular locations^22–25^. One recent prediction methods which is based on protein-protein interactions is introduced by Du *et al*.^25^. In this method, protein-protein interactions are used to improve the results of another prediction method named Hum-mPLoc 2.0^26^.

Here, we present a method based on recommendation systems to predict the locations of a protein. Recommender systems are introduced to recommend products available in e-shops like entertainment items (books, music, videos, images, events and …) that are likely to be of interest to the user^27^. Development of recommender systems is a multi-disciplinary effort, which involves experts from various fields such as artificial intelligence, data mining, statistics, decision support systems and physics^27–29^. In case of a new user, most of the recommender systems are weak to predict proper items. This is called the cold start problem. There are several ways to overcome this problem, for instance content-based methods use tags and categories to make it easier to recommend to new users or users with considerably low information^27,29,30^.

In this paper, we present PMLPR (Protein Multiple Location Prediction based on Recommendation systems) which is a recommendation method based on the bipartite network to predict the SCL of proteins. In our problem, being able to predict the SCL of a new protein is important. Thus, we use the interaction score between proteins in order to overcome the cold start problem.

The PMLPR algorithm, for a given protein, produces a recommendation list of potential locations which are sorted in a descending order with respect to their score, i. e. the location with the higher scores are expected to have a higher chance to be a SCL of that protein. In this algorithm, to construct the bipartite network, the information of SWISS-PROT and the cellular component ontology of GO has been used. The studies show that proteins who interact with each other are more likely be found in the same subcellular localization^31,32^. Therefore, we use the interaction score between two proteins, which is derived from STRING database^33^. STRING database is a web resource of experimentally known and predicted protein-protein interactions.

To evaluate PMLPR method, we compared it with six other state-of-the-art methods, Yloc^34^, WOLF-PSORT^9^, the prediction channel^35^, MDLoc^36^, Du *et al*.^25^ and MultiLoc2-HighRes^37^. Unfortunately, the method introduced by Du *et al*. does not have an online software. Hence, in order to compare with their method, we did the same evaluation test on the same dataset as they mentioned in their publication. The datasets which we used for the evaluation are the set of RAT, FLY and HUMAN proteins and predefined datasets Du *et al*., DBMLoc and Höglund.

## Methods

In this section, we present PMLPR algorithm for protein localization problem. PMLPR is based on one of the existing methods for recommender systems, NBI^28^. In the first part of PMLPR algorithm, the NBI method is used. Then, by applying interaction scores between proteins, PMLPR predicts a list of locations for a protein. In this section, we introduce the NBI method followed by a detailed explanation of our approach.

### NBI

Recommender systems consist of two sets, users and objects. Each user collects a number of objects. The purpose of such systems is to analyze this information and offer new objects to each user. One of the famous recommender systems is NBI algorithm introduced by Zhou *et al*.^28^. NBI is a network-based method which constructs a bipartite network of users and objects. Then, the algorithm performs a resource-allocation process in two steps; First, from objects to users, second from users to objects. The amount of resources after two steps is used to predict new objects for users. Up to now, NBI and its variations are utilized in different research areas. For example, recommending new movies, music and Internet bookmarks to users^28^, predicting new drug targets^38^, and so on.

### PMLPR algorithm

Suppose ${\mathscr{P}}=\{{p}_{1},{p}_{2},\,\ldots ,{p}_{n}\}$ is a set of proteins with known locations and *p* is a new protein that there is no information about its locations. Our algorithm predicts locations for *p* using the information of all proteins in ${\mathscr{P}}$. Suppose ${\mathcal L} =\{{l}_{1},{l}_{2},\ldots ,\,{l}_{m}\}$ be the set of all locations. PMLPR algorithm comprises of four steps as follows:

#### Step 1

A bipartite graph $G=({\mathscr{P}}{\cup }^{} {\mathcal L} ,E)$ is constructed where for ${p}_{i}\in {\mathscr{P}}$ and ${l}_{j}\in  {\mathcal L}$, the edge $e=({p}_{i},{l}_{j})$ belongs to *E* if *p*_*i*_ has already collected *l*_*j*_. In other words, protein *p*_*i*_ belongs to the location *l*_*j*_.

#### Step 2

In this step, the personal recommender matrix R = [*r*_*ij*_] with *n* rows and *m* columns is calculated similar to NBI method. To obtain R, let *A* = [*a*_*ij*_]_*n*×*m*_ be the adjacency matrix of *G* where *a*_*ij*_ = 1 if *p*_*i*_ and *l*_*j*_ are neighbors and *a*_*ij*_ = 0 otherwise. Define *W* = [*w*_*ij*_]_*m*×*m*_ as follows:

1 $${w}_{ij}=\frac{1}{d({l}_{j})}\sum _{t=1}^{n}\frac{{a}_{ti}{a}_{tj}}{d({p}_{t})}$$

In this formula, *d*(*l*_*j*_) and *d*(*p*_*t*_) are the degree of vertices *l*_*j*_ and *p*_*t*_ in G respectively. To obtain the kth row of R, vector $f({p}_{k})={[{a}_{kj}]}_{1\le j\le m}$ is defined as initial resource vector. The kth row of R is calculated by $f({p}_{k})\ast {W}^{T}$, where *W*^*T*^ is the transpose of matrix *W*.

#### Step 3

Let *s*_*ppi*_ denote the interaction score between protein *p* and *p*_*i*_. This score is obtained from STRING database. Define $S(p)=[{s}_{p{p}_{1}},\ldots ,{s}_{p{p}_{n}}]$ and $Pred(p)=S(p)\ast R$. The *i*’th component of *Pred*(*p*) denotes the predicted score of location *l*_*i*_ for protein *p*.

#### Step 4

**Figure 1:**
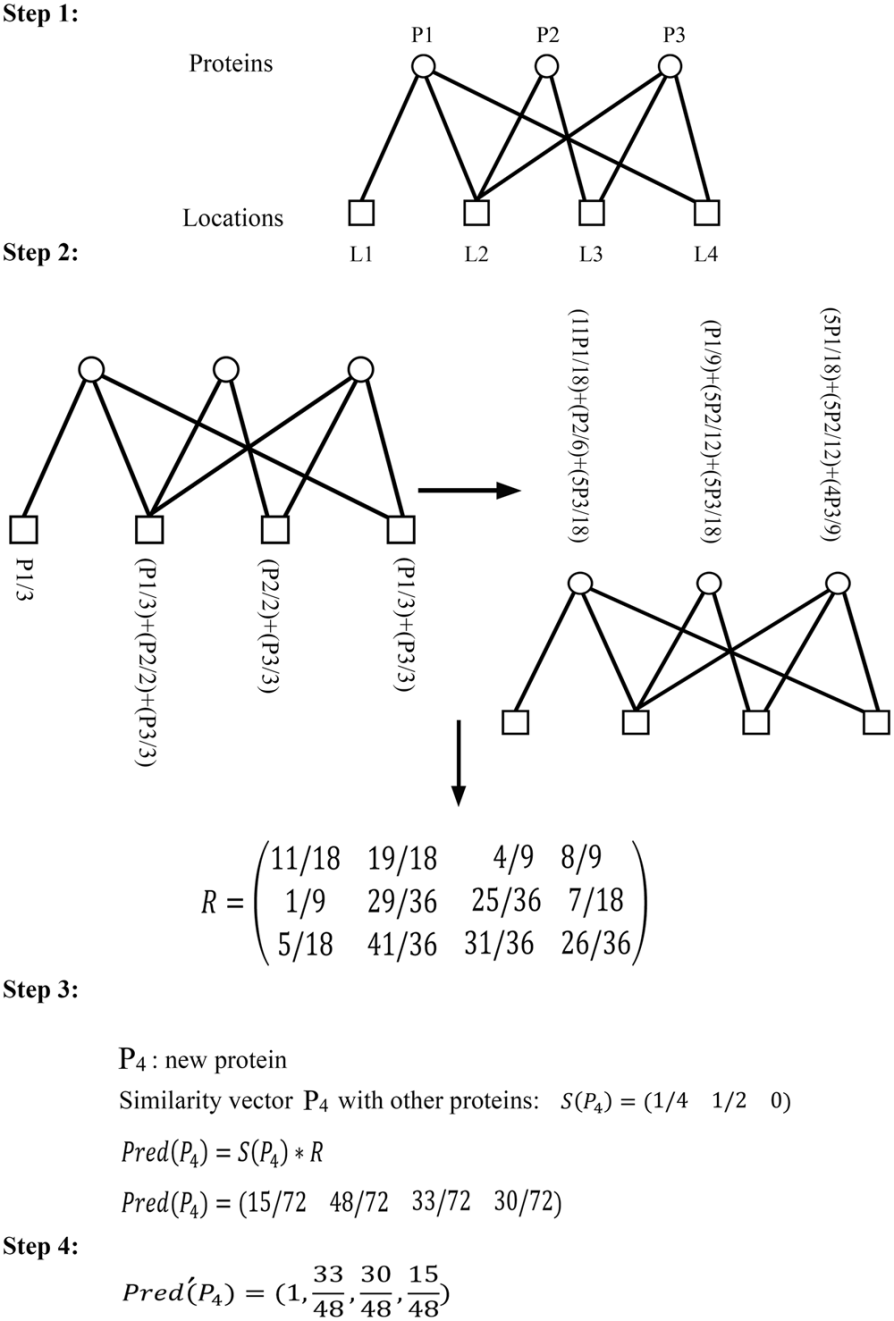
Illustrates all 4 steps of PMLPR algorithm.

In this step, for protein *p*, a set of locations is predicted. To do this, we divide all the scores to the highest score of *Pred*(*p*)and sort them in descending order. We consider these sorted results as $Pred^{\prime} (p)$, which shows the probability of each location for protein *p*. According to a probability threshold, a set of sorted locations can be assigned to protein *p*. A visualization of these 4 steps is shown in Fig. 1. The first 2 steps demonstrate the resource-allocation process in a bipartite network. In step 3, an interaction vector *S*(*p*_4_) is used to calculate the *Pred*(*p*_4_). In step 4, $Pred^{\prime} (p)$ is calculated. A desired threshold can be applied and a list of locations is predicted.

### Data availability

http://facultymembers.sbu.ac.ir/eslahchi/en/portfolio-items/subcellular-protein-localization/.

## Results

To evaluate PMLPR algorithm, six datasets containing, RAT, FLY, HUMAN proteins, Du *et al*., DBMLoc^39^ and Höglund^37^ are exploited. The results of PMLPR algorithm are compared to the result of six different state-of-the-art algorithms, Yloc, WOLF-PSORT, prediction channel of compartment, MDLoc and Du *et al*.

### Protein datasets

The set of RAT, FLY and HUMAN proteins are obtained from UniProtKB/Swiss-Prot release 2017^18,19^. Only the reviewed and manually annotated information is considered which is known as Swiss-Prot dataset. The RAT, FLY and HUMAN contain 7928, 2850 and 20203 proteins, respectively. Meanwhile, CD-HIT^40^ is used to reduce the redundancy of the protein dataset. Proteins with 35% similarity and above are eliminated from the dataset. After applying CD-HIT, the number of proteins in RAT, FLY and HUMAN are 5301, 2474 and 13250 respectively. Then, the protein-location dataset is updated, and PMLPR results on this dataset is calculated.

In order to compare PMLPR with other cutting-edge prediction tools, three other datasets have been used. The first one, is introduced by Du *et al*. In this dataset, all the HUMAN proteins were obtained from BioGRID dataset, mapped into 18036 proteins in UniProt dataset.

Two other benchmark datasets are DBMLoc and Höglund. DBMLoc contains 10470 multiple subcellular localization-annotated entries, which all these protein entries are cross-referenced to GO-annotations and SwissProt^39^. DBMLoc contains 6 subcellular localizations, Cytoplasm, Mitochondrion, Nucleus, Plasma Membrane, Secreted, ER. Höglund contains 5959 protein entries and 11 subcellular localizations, Chloroplast, Cytoplasmic, ER, Extracellular, Golgi, Lysosomal, Mitochondrial, Nuclear, Proxisomal, Plasma-membrane, vacoular. In Höglund, BLASTClust has been used to cluster the sequences using 30% threshold for pairwise sequence identity in animal and fungal proteins and 40% threshold in plant proteins^37^.

### Locations datasets

For each protein, a set of subcellular locations are obtained from the cellular_ component dag of GO (Gene Onthology) release 2015. Moreover, the subcellular locations [CC] derived from Swiss-Prot are considered as well. For all RAT, FLY and HUMAN datasets, 9 subcellular locations, including Cytoplasm, Cytoskeleton, ER (Endoplasmic reticulum), ExR (Extracellular region), Membrane, Mit (Mitochondrion), Nucleus, GA (Golgi apparatus) and Peroxisome are considered. Most of the Intermembrane/Transmembrane proteins are identical among Plasma Membrane, ER membrane, etc. In this study, we consider all as Membrane.

In order to compare our results with Du *et al*., eleven subcellular locations have been considered, including Cell membrane, Cytoplasm, ER, Extracellular region, Golgi Apparatus, Mitochondrion, Nucleus, Peroxisome, Lysosome, Endosome and Microsome. For a protein, if a subcellular location has been marked as “Probable”, “By Similarity” or “Potential”, the subcellular location has been discarded.

### Evaluation Method

To assess the performance of PMLPR against other algorithms, four different measurements are employed.

#### Measure 1

Measurements commonly used in many evaluation methods are Precision, Recall and F-measure. The Precision calculates the fraction of retrieved instances that are relevant and Recall calculates the fraction of relevant instances retrieved.

2 $${\rm{Precision}}=\frac{1}{|D|}\,\sum _{p}\frac{|l^{\prime} (p){\cap }^{}l(p)|}{|l^{\prime} (p)|}$$

3 $${\rm{Recall}}=\frac{1}{|D|}\sum _{p}\frac{|l^{\prime} (p){\cap }^{}l(p)|}{|l(p)|}$$

4 $${\rm{F}} \mbox{-} {\rm{m}}{\rm{e}}{\rm{a}}{\rm{s}}{\rm{u}}{\rm{r}}{\rm{e}}=\frac{2\ast {\rm{P}}{\rm{r}}{\rm{e}}{\rm{c}}{\rm{i}}{\rm{s}}{\rm{i}}{\rm{o}}{\rm{n}}\ast {\rm{R}}{\rm{e}}{\rm{c}}{\rm{a}}{\rm{l}}{\rm{l}}}{{\rm{P}}{\rm{r}}{\rm{e}}{\rm{c}}{\rm{i}}{\rm{s}}{\rm{i}}{\rm{o}}{\rm{n}}+{\rm{R}}{\rm{e}}{\rm{c}}{\rm{a}}{\rm{l}}{\rm{l}}}$$

where |D| denotes the number of proteins. For a protein, *l*(*p*) = {*x*_1*p*_, … *x*_*kp*_} and $l^{\prime} (p)=({y}_{1p},\ldots {y}_{tp})$ be the set of locations, which protein *p* localized according to the dataset and the order set of locations that a prediction algorithm predicts for protein *p*, respectively. In this evaluation, we do not consider the order of locations predicted for each protein. Using this approach, we globally evaluate the performance of an algorithm regardless of the order of locations introduced for a protein. For example, if the order set (nucleus, cytoplasm) is introduced for protein *p*, Precision does not consider the order of locations and there is no significant difference between (nucleus, cytoplasm) and (cytoplasm, nucleus). However, with more reliability the algorithm suggest that the protein p is located in nucleus in the first prediction and cytoplasm in the second prediction. In order to consider this difference, we introduce an extra measurement. Let the intersection of *l*(*p*) and $l^{\prime} (p)$ be the order set, $l(p){\cap }^{}l^{\prime} (p)=({y}_{{i}_{1}p},{y}_{{i}_{2}p},\ldots ,{y}_{{i}_{r}p})$.

Define:

5 $$Pr{e}_{p}=\,\frac{(t-{i}_{1}+1)+(t-{i}_{2}+1)+\ldots +(t-{i}_{r}+1)}{{\rm{\Delta }}(t,k)}$$

where:

6 $${\rm{\Delta }}(t,k)=\{\begin{array}{rr}t+(t-1)+\ldots +(t-k+1), & t\ge k\\ t+\ldots +1, & t < k\end{array}$$

7 $${\rm{O}}{\rm{r}}{\rm{d}}{\rm{e}}{\rm{r}}{\rm{e}}{\rm{d}}{\rm{P}}{\rm{r}}{\rm{e}}{\rm{c}}{\rm{i}}{\rm{s}}{\rm{i}}{\rm{o}}{\rm{n}}=\frac{1}{|D|}\sum _{p\in D}Pr{e}_{p}$$

8 $${{\rm{F}}}_{{\rm{ordered}}} \mbox{-} \mathrm{measure}=2\ast \frac{OrderedPrecision\ast Recall}{OrderedPrecision+Recall}$$

Since, Precision and Ordered Precision, reflect the size of the prediction and the order of the prediction respectively, we introduced:

9 $$MP=\frac{Precision+OrderedPrecision}{2}$$

which is the mean of the two measurements Precision and Ordered Precision.

Finally, *F*_*MP*_-*measure* is defined as follows:

10 $${{\rm{F}}}_{{\rm{MP}}} \mbox{-} \mathrm{measure}=2\ast \frac{MP\ast Recall}{MP+Recall}$$

#### Measure 2

The second measurement is introduced by Simha *et al*.^36^. For each location c, Pre_c_ and Rec_c_ are defined as follow:

11 $${{\rm{Pre}}}_{{\rm{c}}}=\frac{1}{|\{p|c\in l^{\prime} (p)\}|}\sum _{p|c\in l^{\prime} (p)}\frac{|l^{\prime} (p){\cap }^{}l(p)|}{|l^{\prime} (p)|}$$

12 $${{\rm{Rec}}}_{{\rm{c}}}=\,\frac{1}{|\{p|c\in l(p)\}|}\sum _{p|c\in l(p)}\frac{|l^{\prime} (p){\cap }^{}l(p)|}{|l(p)|}$$

In this part, prec_c_ and Rec_c_ obtain the Precision and Recall of an algorithm for each location c. Moreover, Simha *et al*. considered F_1_-score_c_, the harmonic mean of Precision and Recall for each location c. Furthermore, the average F_1_-score for all locations are calculated as follow:

13 $${{\rm{F}}}_{1}-{{\rm{s}}{\rm{c}}{\rm{o}}{\rm{r}}{\rm{e}}}_{{\rm{c}}}=\frac{2\ast {{\rm{P}}{\rm{r}}{\rm{e}}}_{{\rm{c}}}\ast {{\rm{R}}{\rm{e}}{\rm{c}}}_{{\rm{c}}}}{{{\rm{P}}{\rm{r}}{\rm{e}}}_{{\rm{c}}}+{{\rm{R}}{\rm{e}}{\rm{c}}}_{{\rm{c}}}}$$

14 $${{\rm{F}}}_{1}-{\rm{score}}=\frac{1}{|C|}\sum _{c}{{\rm{F}}}_{1}{-\mathrm{score}}_{{\rm{c}}}$$

#### Measure 3

The third measurement is introduced by Du *et al*.^25^. They introduced 5 statistical measures, Recall (AIM), Precision (CVR), ACC′, ATR and AFR. The first two statistical measures, Recall and Precision are introduced in Measure 1. ACC′, ATR and AFR are accuracy, absolute true-rate and absolute false-rate, respectively. They can be formulated as followed:

15 $${\rm{ACC}}^{\prime} =\frac{1}{|D|}\sum _{p}\frac{|l^{\prime} (p){\cap }^{}l(p)|}{|l^{\prime} (p){\cup }^{}l(p)|}$$

16 $${\rm{ATR}}=\frac{1}{|D|}\sum _{p}\delta [l^{\prime} (p),l(p)]$$

17 $${\rm{AFR}}=\frac{1}{|D|\ast |C|}\,\sum _{p}[|l^{\prime} (p){\cup }^{}l(p)|-|l^{\prime} (p){\cap }^{}l(p)|]$$

Where |*C*| is the number of subcellular locations. and

18 $$\delta [l^{\prime} (p),l(p)]=\{\begin{array}{ll}1, & l^{\prime} (p)=l(p)\\ 0,\, & otherwise\end{array}.$$

#### Measure 4

The forth measurement is ACC(accuracy), which is slightly different from ACC′ ACC can be formulated as followed:

$${\rm{ACC}}=\frac{1}{|D|}\sum _{p}\frac{|l^{\prime} (p){\cap }^{}l(p)|+|C-(l(p){\cup }^{}l^{\prime} (p))|}{|C|}$$

### Performance Evaluation

As Chuo *et al*. mentioned in their publication^41^, in order to compare the results of various prediction algorithms, there are three methods, Independent dataset, k-fold cross-validation and jackknife test (one-leave-out cross-validation). Since the proteins of the independent test should be apart from the training set, there is a major problem to choose the independent dataset. How to select this independent dataset can completely change the final results. It is axiomatic that this method is not efficient for our comparison.

On the other hand, in the k-fold cross-validation test, the benchmark should be divided into k class of data. As Chuo *et al*. mentioned in their publication^41^, the number of possible selections to divide a benchmark into k classes is an immense number. Hence, selecting one of the divisions cannot be a fair demonstration of the performance of the algorithm.

Jackknife method considers each protein as a test case. In fact, in this method each protein moves between the train and test datasets. Moreover, this method is more efficient in memory usage. For these testimonies, jackknife method does not have the mentioned problems and it truly fits our problem. Thus in this paper, jackknife method is mainly used due to representing the performance of the algorithms impartially. Plus, we applied k-fold cross-validation method for more affirmation. In order to evaluate the accuracy of the algorithm, per each test protein, a list of locations is predicted according to the training dataset.

In PMLPR algorithm, for each prediction, we introduce a reliability threshold. According to this threshold, a set of sorted locations can be assigned for each protein. This threshold is used to exclude predictions with low reliability score. It is possible for the users to change this reliability threshold in the online version of PMLPR algorithm. For example, if the reliability threshold of 80% is considered for sample protein P35213, PMLPR’s sorted result will be $l^{\prime} (p)$ = (cytoplasm, membrane), and if the reliability threshold of 30% is considered, the sorted list for this protein will be $l^{\prime} (p)$ = (cytoplasm, membrane, nucleus). In this study, in order to compare the results of our algorithm with the other state-of-the-art methods, we consider the reliability threshold of 30%.

#### Jackknife Test

Table 1 depicts the comparison between the results of PMLPR algorithm with the results of WP (WOLF-PSORT) and PC (prediction channel of compartment) on three species RAT, FLY and HUMAN.

**Table 1:** Comparison of PMLPR with 2 other methods based on Measure 1(PC = Prediction channel, WP = WOLF-PSORT).

		Recall	Precision	Ordered-Precision	MP	F-measure	F_ordered_-measure	F_MP_-measure
**RAT**	**PMLPR**	**0.846**	**0.903**	**0.951**	**0.927**	**0.873**	**0.895**	**0.884**
	**WP**	0.781	0.626	0.809	0.717	0.694	0.794	0.747
	**PC**	0.729	0.575	0.758	0.666	0.642	0.743	0.696
**FLY**	**PMLPR**	**0.912**	**0.499**	**0.824**	**0.661**	**0.645**	**0.865**	**0.766**
	**WP**	0.596	0.263	0.486	0.374	0.364	0.535	0.459
	**PC**	0.615	0.255	0.508	0.381	0.36	0.556	0.47
**HUMAN**	**PMLPR**	**0.935**	0.302	0.706	0.504	0.45	**0.804**	0.654
	**WP**	0.796	**0.427**	0.751	0.589	0.555	0.772	0.677
	**PC**	0.81	**0.427**	**0.776**	**0.601**	**0.559**	0.792	**0.69**

The predefined Measure 1 (Recall, Precision, OrderedPrecision, MP, F-measure, F_ordered_-measure and F_MP_-measure) is used to compare the performances of algorithms in Table 1. This table reveals that on RAT and FLY proteins, PMLPR dramatically improved the results in all tests. In RAT and FLY, PMLPR improved the performance by at least 0.1 and 0.3, respectively. For instance, PMLPR improved the F_ordered_-measure and F-measure on RAT proteins by 0.1 and 0.18 with respect to the results of WP, which has the best result between the other methods. As can be seen from Table 1, on Fly dataset, PMLPR has a noticeable improvement in all tests. For example, PMLPR bucked up the F_ordered_-measure results for 0.31. Albeit, Table 1 demonstrates comparable results on HUMAN dataset. On HUMAN, PMLPR indicate the best F_ordered_-measure, PC shows the highest F-*measure* and F_MP_-measure. To sum up, in most cases, Table 1 shows that the Recall, Precision, OrderedPrecision, F-measure, F_ordered_-measure and F_MP_-measure values have been increased significantly by PMLPR algorithm with respect to other algorithms, which implies the efficiency of our method.

The other comparison used to evaluate the performance of PMLPR is the one introduced by Simha *et al*.^36^ and we defined it in section 3, measure 2. Table 2 shows the result of this comparison (F_1_-score_c_) between different algorithms, per each 9 locations on RAT, FLY and HUMAN proteins.

**Table 2:** F1-scorec results per 9 locations: Cytoplasm, Cytoskeleton, ER(Endoplasmic Reticulum), ExR(Extracellular Region), Membrane, Mit(Mitochondrion), Nu-cleus, GA(Golgi Apparatus),Peroxisome.

		Cytoplasm	Cytoskeleton	ER	ExR	Membrane	Mit	Nucleus	GA	Peroxisome
**RAT**	PMLPR	**0.591**	**0.557**	**0.49**	**0.469**	**0.574**	**0.513**	**0.58**	**0.543**	**0.427**
	WP	0.432	0.332	0.336	0.391	0.434	0.381	0.456	0.299	0.338
	PC	0.443	0.336	0.347	0.39	0.434	0.387	0.464	0.214	0.363
**FLY**	PMLPR	**0.576**	**0.516**	**0.418**	**0.46**	0.554	**0.438**	**0.572**	—	—
	WP	0.45	0.388	0.31	0.389	**0.581**	0.382	0.56	—	0.31
	PC	0.468	0.398	0.312	0.393	0.52	0.382	0.567	—	**0.333**
**HUMAN**	PMLPR	**0.498**	0.332	0.349	0.416	0.493	**0.452**	0.51	**0.379**	**0.471**
	WP	0.495	**0.405**	**0.361**	**0.435**	**0.585**	0.416	**0.553**	0.318	0.387
	PC	0.487	0.382	0.342	0.4	0.494	0.363	0.52	0.309	0.345

As it can be distinguished from Table 2, PMLPR has the best performance on RAT and FLY proteins and on HUMAN the results are quite competing, WP has the best performance in five of the locations and PMLPR has the best performance on four of the locations. Based on the results of Table 2, PMLPR has the best performance on all locations or a score close to the best performance. Overall, it can be said that PMLPR has acceptable performance on all locations.

Table 3 illustrates the F_1_-score, the average F_1_-score_c_ over all 9 locations. This table shows that, PMLPR has the best overall performance on RAT and FLY, competing results on HUMAN.

**Table 3:** F1-score results over all 9 locations.

	PMLPR	WP	PC
RAT	**0.527**	0.377	0.375
FLY	**0.393**	0.374	0.374
HUMAN	0.433	**0.439**	0.404

Overall, all these tests depicted the efficiency of PMLPR method. PMLPR has a significant improvement on RAT and FLY datasets. Furthermore, on HUMAN dataset, PMLPR has almost the same performance as other reported state-of-the-art methods.

Whereas Du *et al*. did not provide their software, we were unable to obtain their result for any protein to perform Measure 1 and Measure 2. In order to compare our method with them, we applied the same evaluation test as they performed. Hence, we would be able to use their result in our comparison. The results are shown in Table 4. Since we used a threshold of 0.3 in this test, PMLPR has wider range of predictions. Consequently, this would cause a higher recall and Absolute False-Rate(AFR) and lower precision, ACCuracy(${\rm{ACC}}^{\prime}$) and Absolute True-Rate(ATR). However, by increasing the threshold to 0.7 the Recall, Precision, ${\rm{ACC}}^{\prime}$, ATR and AFR would be 0.715, 0.634, 0.609, 0.568 and 0.081 respectively. Plus, Du *et al*. just worked on HUMAN proteins, so we could not test their algorithm on RAT and FLY proteins. However, we had competing results on HUMAN proteins.

**Table 4:** Result of Measure 3 on Human proteins.

	Recall	Precision	ACC′	ATR	AFR
PMLPR 0.3	**0.915**	0.433	0.421	0.387	0.118
PMLPR 0.7	0.755	0.63	0.649	**0.568**	0.081
YLoc	0.724	0.61	0.598	0.474	0.084
Du *et al*.	0.798	**0.749**	**0.7**	0.56	**0.065**

#### Cross-validation test on DBMLoc and Höglund datasets

In order to further evaluate PMLPR on other species based on the existing datasets, two of the well stablished datasets, DBMLoc and Höglund has been used. A similar 5-fold cross-validation test as the one performed by Zhou *et al*. in their publication has been used. This 5-fold cross-validation test has been repeated thirty times, and the average outcome is represented in Table 5. The ACC which is used in this evaluation is introduced in measure 4. While using these multi-species datasets, we faced the problem of building the similarity vector between proteins. It is trivial that there could be no protein-protein interaction between two proteins from two different species. DBMLoc and Höglund contain different proteins from different species, and in some species these two datasets have very few proteins. As mentioned in step 3 in section 2.2, we used the protein-protein interaction dataset, STRING, in order to build the similarity vector between proteins. Thus, the similarity vector built based on STRING was too sparse, and insufficient. To overcome this problem, we decided to use the sequence similarity of these proteins. For this purpose, a smith-waterman^42^ sequence alignment between proteins has been applied, to obtain the protein-protein similarity for these two datasets.

**Table 5:** Average results of ACC and F-Measure, on 30 runs of 5-fold cross-validation results on DBMLoc and Höglund.

	ACC/F-measure	
	DBMLoc	Höglund
PMLPR	**0.72**/0.67	**0.64**/0.38
YLoc+	0.64/**0.68**	0.53/0.37
MultiLoc2-HighRes	—	0.57/**0.41**

As can be seen from Table 5, PMLPR has the highest ACC in both datasets. In case of F-measure, PMLPR results on both DBMLoc and Höglund datasets are quiet comparable.

#### Cross-validation test on RAT, FLY and HUMAN datasets

We performed a 10-fold cross-validation test on PMLPR results. Since the implementation of the other existing methods are not available, we were unable to make change to the training data to compare the methods by 10-fold cross validation test. Besides, as the authors do not provide all the details of their implementations in their papers, re-implementing these methods may cause in unreliable results. Hence, we performed a 10-fold cross validation on PMLPR results for thirty times. The average outcome of this test, demonstrates that there is a negligible difference between the results of jackknife and cross-validation test. For instance, Table 6 and Table 7 display the average results of 10-fold cross-validation test on RAT, FLY and HUMAN proteins. As can be seen from these two tables, the results of the 10-fold cross-validation test are similar to the results of jackknife test. Therefore, we can consider jackknife as a reliable evaluation method for this problem.

**Table 6:** Average results of Measure 1, on 30 runs of 10-fold cross-validation results on RAT, FLY and HUMAN.

	Recall	Precision	OrderedPrecision	MP	F-measure	F_ordered_-measure	F_MP_-measure
RAT	0.873	0.88	0.86	0.87	0.876	0.867	0.871
FLY	0.895	0.45	0.799	0.625	0.599	0.844	0.736
Human	0.912	0.25	0.652	0.451	0.392	0.76	0.603

**Table 7:** Average F1-scorec results per 9 locations: Cytoplasm, Cytoskeleton, ER(Endoplasmic Reticulum), ExR(Extracellular Region), Membrane, Mit(Mitochondrion), Nucleus, GA(Golgi Apparatus),Peroxisome on 30 runs of 10-fold cross-validation results on RAT, FLY and HUMAN.

	Cytoplasm	Cytoskeleton	ER	ExR	Membrane	Mit	Nucleus	GA	Peroxisome
RAT	0.571	0.502	0.451	0.416	0.517	0.472	0.526	0.484	0.343
FLY	0.527	0.497	0.391	0.42	0.528	0.416	0.534	0	0
HUMAN	0.467	0.29	0.326	0.381	0.436	0.412	0.475	0.314	0.412

#### Specific proteins

Table 8 shows 8 proteins with their subcellular locations and Gene Ontology information. These proteins are believed to be important in different cancers^43–49^. We have selected these proteins in order to have a transpicuous comparison between PMLPR and the 4 other methods. Table 9 demonstrates the results of each method for these 8 specific proteins. Since Cytosol and Cytoplasm are two very similar locations we decided to consider them as a unified location and named it Cyt in this table. It can be seen that PMLPR predicts plenty of locations for each of the proteins, however not all the methods cover sufficient number of the predictions for each protein. For instance, Yloc has only one prediction for 7 out of 8 proteins, and MDLoc has at most two predictions for each protein. This can be considered as a weak point of these two well-known methods. Considering the protein O43683 (gene name: BUB1), Nucleus, Cyt and Membrane are the pre-known locations for this protein, based on Swiss-Prot and Gene Ontology. For O43683, PMLPR predicts all the 3 locations (Nucleus, Cyt and Membrane) correctly, while, YLoc predicts only one of the locations (Nucleus), MDLoc, WP and PC predict 2 of the locations. MDLoc, WP and PC predict Cyt and Nucleus. For another example, we can consider protein Q43663 (gene name: PRC1), Nucleus, Cyt, Membrane and Cytoskeleton are the pre-known locations for this protein. For Q43663, PMLPR predicts 4 different locations (Cyt, Nucleus, Membrane and Cytoskeleton), where all of the 4 predictions are correct, YLoc predicts only 1 location, Nucleus. MDLoc, WP and PC predict two of the locations, Cyt and Nucleus. On the other hand, PMLPR has some limitations as well. Consider protein Q569k4 (gene name: ZNF385B) whose pre-known location is Nucleus. For this protein, PMLPR predicts 4 different locations (Membrane, Cyt, Nucleus and Mitochondrion) where only Nucleus in the third place is correct. While YLoc, WP and PC predict Nucleus accurately and MDLoc has two predictions for this protein (Cyt and Nucleus). Each of the existing methods have their own limitations and weak points. Especially on HUMAN proteins, the results of these methods are closely comparable.

**Table 8:** 8 selected proteins with their subcellular location and gene ontology information (from UniProt).

Gene Name	Entry	Subcellular location [CC]	Gene ontology (cellular component)
TK1	P04183	Cytoplasm	Cytosol
ZNF385B	Q569K4	Nucleus	Nucleus
ELOVL1	Q9BW60	Membrane, ER	Membrane, ER
TBX3	O15119	Nucleus	Nucleus
BUB1	O43683	Nucleus	Cytoplasm, Cytosol, Membrane
PRC1	O43663	Cytoplasm, Nucleus, Cytoskeleton	Cytoplasm, Nucleus, Cytosol, Membrane, Cytoskeleton
CCNE2	O96020	Nucleus	Cytosol
CHAF1B	Q13112	Nucleus, Cytoplasm	Nucleus, Cytoplasm

**Table 9:** Results of each method for 8 selected protein (Nuc = Nucleus, Cyt = Cytoplasm\Cytosol, Mem = Membrane, Mit = Mitochondrion, ER = Endoplasmic Reticulum, ExR = Extracellular Region, Per = Peroxisome, GA = Golgi apparatus).

Gene Name	PMLPR	MDLoc	Yloc	WP	PC
TK1	Nuc, **Cyt**, Mem	**Cyt**	**Cyt**	**Cyt**, Nuc, Mit, ExR, Per	**Cyt**, Nuc, Mit, ExR, Per
ZNF385B	Mem, Cyt, **Nuc**, Mit	Cyt, **Nuc**	**Nuc**	**Nuc**	**Nuc**
ELOVL1	**Mem**, Cyt, **ER**	Cyt, **Mem**	**Mem, ER**	**Mem, ER**	**Mem, ER**
TBX3	**Nuc**, Mem, Cyt	Cyt,**Nuc**	**Nuc**	**Nuc**, Cyt, Mit	**Nuc**, Cyt, Mit
BUB1	**Nuc, Cyt, Mem**	**Cyt, Nuc**	**Nuc**	**Nuc, Cyt**	**Nuc, Cyt**
PRC1	**Cyt, Nuc, Mem, Cytoskeleton**	**Cyt, Nuc**	**Nuc**	**Nuc, Cyt**	**Nuc, Cyt**
CCNE2	**Nuc, Cyt**	**Nuc**	**Nuc**	**Nuc, Cyt**, Mit	**Nuc, Cyt**, Mit
CHAF1B	**Nuc, Cyt**, Mem	**Cyt**	**Cyt**	**Nuc, Cyt**	**Nuc, Cyt**

## Discussion

We presented an efficient protein localization method using personal recommender systems and protein-protein interactions. Using such approach for protein localization problem is the main contribution of this paper. The results demonstrate the utility of using recommender systems and protein-protein interactions in the prediction process. PMLPR not only improves the results, but also has a fast algorithm. The related algorithm is implemented using C++/R languages.

To the best of our knowledge, there are no available subcellular prediction software using protein-protein interactions, especially on HUMAN proteins. PMLPR software is available online and it is useable by biologist and other scientist.

## Future Works

NBI is one of the basic recommender systems, there are more complex recommender systems, such as content-based methods^30^, collaborative filtering^50^, matrix factorization^51^ and etc. These methods can be applied in this problem, and they may improve the prediction results.

In recent methods such as MDLoc, the interdependency of the locations has been taken into the account, because some of the locations have high interaction with each other and many proteins travel between these locations constantly. These interdependencies can be used in the future studies of this problem. Moreover, a fusion between our method and the other best existing methods will improve the results.
